# *β*3-Adrenoreceptors Control Mitochondrial Dormancy in Melanoma and Embryonic Stem Cells

**DOI:** 10.1155/2018/6816508

**Published:** 2018-11-13

**Authors:** Maura Calvani, Lorenzo Cavallini, Annalisa Tondo, Valentina Spinelli, Luisa Ricci, Amada Pasha, Gennaro Bruno, Daniela Buonvicino, Elisabetta Bigagli, Marina Vignoli, Francesca Bianchini, Laura Sartiani, Maura Lodovici, Roberto Semeraro, Filippo Fontani, Francesco De Logu, Massimo Dal Monte, Paola Chiarugi, Claudio Favre, Luca Filippi

**Affiliations:** ^1^Oncohematology Unit, Department of Pediatric Oncology, A. Meyer Children's University Hospital, Florence, Italy; ^2^Department of Experimental and Clinical Medicine, University of Florence, Florence, Italy; ^3^Department of NEUROFARBA, Section of Pharmacology and Toxicology, University of Florence, Florence, Italy; ^4^Department of Health Sciences, University of Florence, Florence, Italy; ^5^Department of Experimental and Clinical Biomedical Sciences, University of Florence, Florence, Italy; ^6^Department of Biology, University of Pisa, Italy; ^7^Neonatal Intensive Care Unit, Medical Surgical Fetal-Neonatal Department, A. Meyer Children's University Hospital, Florence, Italy

## Abstract

The early phases of embryonic development and cancer share similar strategies to improve their survival in an inhospitable environment: both proliferate in a hypoxic and catecholamine-rich context, increasing aerobic glycolysis. Recent studies show that *β*3-adrenergic receptor (*β*3-AR) is involved in tumor progression, playing an important role in metastasis. Among *β*-adrenergic receptors, *β*3-AR is the last identified member of this family, and it is involved in cancer cell survival and induction of stromal reactivity in the tumor microenvironment. *β*3-AR is well known as a strong activator of uncoupling protein 1 (UCP1) in brown fat tissue. Interestingly, *β*3-AR is strongly expressed in early embryo development and in many cancer tissues. Induction of uncoupling protein 2 (UCP2) has been related to cancer metabolic switch, leading to accelerated glycolysis and reduced mitochondrial activity. In this study, for the first time, we demonstrate that *β*3-AR is able to promote this metabolic shift in both cancer and embryonic stem cells, inducing specific glycolytic cytoplasmic enzymes and a sort of mitochondrial dormancy through the induction of UCP2. The *β*3-AR/UCP2 axis induces a strong reduction of mitochondrial activity by reducing ATP synthesis and mitochondrial reactive oxygen species (mtROS) content. These effects are reverted by SR59230A, the specific *β*3-AR antagonist, causing an increase in mtROS. The increased level of mtROS is neutralized by a strong antioxidant activity in embryonic stem cells, but not in cancer stem cells, where it causes a dramatic reduction in tumor cell viability. These results lead to the possibility of a selective antitumor therapeutic use of SR59230A. Notably, we demonstrate the presence of *β*3-AR within the mitochondrial membrane in both cell lines, leading to the control of mitochondrial dormancy.

## 1. Introduction

Both fetal development and tumor growth, despite, respectively, physiological and pathological events with feedback far away from each other, are triggered by heterogeneous interrelated pathways. Embryo and cancer, among hypoxic active stromal tissues and high stem cell potential [1], share a high aerobic glycolytic rate [2, 3] and a catecholamine-rich environment [4, 5]. Aerobic glycolysis occurs in the early preimplantation of mammalian embryo [6, 7], during early pregnancy [8], and in tumors [4], by increasing the consumption of glucose and production of lactate. Aerobic glycolysis, which converts glucose to lactate, is the major source of energy of these cells, instead of mitochondrial oxidative phosphorylation [4, 9, 10]. Both cancer cells and embryos need to increase the uptake of glucose, the expression of glycolytic enzymes, and the export of lactate to obtain energy for growth [6–11]. Glycolytic metabolism confers a proliferative advantage to both tumor cells and embryos, since this metabolism produces a large number of useful intermediates to secondary biosynthetic pathways [5, 12].

Several studies suggest that stress-related catecholamine release accelerates cancer progression, so the targeting of *β*-adrenergic receptors (*β*-ARs) has been proposed as a potential therapeutic approach to cancer and, in particular, melanoma [13, 14]. At the beginning, attention was mainly focused on *β*2-AR [15], but, more recently, *β*3-AR was found overexpressed in tumors, particularly in melanoma cell lines and tumor microenvironments [16–18]. Currently, it is known that *β*3-AR expression correlates with cancer progression, angiogenesis, and tumor stromal cell reactivity [16–18]. Interestingly, high expression of *β*3-AR has also been revealed in the pregnant myometrium [19] and during early embryo development [20, 21], confirming the similarities between the environments surrounding tumor and embryo.

*β*3-AR is well known to control thermogenesis by activating uncoupling protein 1 (UCP1). At low temperature, a signal is transmitted to brown adipocytes by catecholamine release and *β*3-AR activation, resulting in increased UCP1 expression and activity. The UCPs are members of the mitochondrial anion carrier family. While UCP1 is predominantly expressed in brown adipocyte tissue [22], UCP2 is ubiquitously expressed in cancer derived from different tissues [23, 24]. Interestingly, UCP2 appears to be highly expressed in cancer cells (such as leukemia and pancreatic cancer) and in nondifferentiated cells with low amounts of mitochondrial tissues, which rely on glycolysis rather than oxidative phosphorylation for their energy production [25–28].

In this study, we investigated the role of the *β*3-AR/UCP2 axis on the regulation of the Warburg metabolism in cancer stem cells (CSC) and in mouse embryonic stem cells (ESC).

## 2. Materials and Methods

### 2.1. Cell Culture and ES Cell Differentiation

Melanoma cells (A375) and mouse ESC (CGR8) were purchased from the European Collection of Cell Cultures (ECACC, Wiltshire, UK).

A375 cells were cultured in high-glucose DMEM containing 10% FBS, while CGR8 were cultured in a propagation medium containing a BHK21 medium (Gibco), 1% nonessential amino acids (Gibco), 10^−3^ M sodium pyruvate (Gibco), 10^−7^ M beta-mercaptoethanol (Sigma), 2 × 10^−3^ M glutamine (Sigma) 1% penicillin-streptomycin (Gibco), 10% fetal bovine serum (Gibco), and 1000 U/ml recombinant mouse leukemia inhibitory factor (Chemicon International) in a humidified 5% CO_2_ atmosphere at 37°C [29]. Differentiation of ES cells was performed by the hanging drop method [30]. Briefly, embryoid bodies (EBs) were formed for 2 days in hanging drops containing 450–600 cells/20 *μ*l of a differentiation medium, whose composition was similar to that used for cell propagation except for fetal bovine serum (20%, HyClone) and without recombinant mouse leukemia inhibitory factor. After 4 days in suspension, EBs were plated on gelatin-coated plates. Starting from day 8 of differentiation, the number of beating EBs was counted by phase-contrast microscopy.

### 2.2. Cell Treatments

A375 cells were grown to 70% confluence in complete medium DMEM high-glucose for 48 h. Then the medium was removed, and after washing in PBS solution, cells were serum-starved overnight with a starvation medium, without FCS, in order to promote the cells' entry into a quiescent G0 phase, thereby better evaluating cells' responsiveness to exogenous treatments. After 24 h, cells were treated with a single dose of BRL, a *β*3-adrenergic agonist, and after 30 minutes with the *β*3-antagonist SR59230A and with genipin, UCP2's antagonist.

### 2.3. Melanosphere Formation Assay

A375 cells were incubated for 72 h with a conditioned medium (CM) and then detached using Accutase (Sigma). Single A375 cells were plated at 150 cells/cm^2^ on a low-attachment 100 mm plate in a Dulbecco's modified Eagle's medium/F12 supplemented with B27 and N2 media, 5 g/ml insulin, 20 ng/ml fibroblast GF-2, and 20 ng/ml epidermal GF. A375 cells were grown under these conditions for 15–20 days and formed nonadherent P0 spheres termed prostaspheres. For the evaluation of self-renewal, a single prostasphere was dissociated in single cells with Accutase, and a dilution of one cell per well into 96-well low-attachment plates was performed in order to isolate individual P1 prostaspheres. Single-cell cloning was confirmed by microscopic analysis, and single clones were counted.

### 2.4. Western Blot Analysis

The buffer used was RIPA lysis buffer (50 mM Tris-HCl, pH 7.5, 150 mM NaCl, 1% Triton X-100, 2 mM EGTA, 1 mM sodium orthovanadate, 1 mM phenylmethanesulphonyl fluoride, 10 mg/ml aprotinin, and 10 mg/ml leupeptin). Twenty micrograms of total proteins was loaded on SDS-PAGE, separated, and transferred onto nitrocellulose. The immunoblots were incubated in 3% bovine serum albumin, 10 mmol/l Tris-HCl (pH 7.5), 1 mmol/l EDTA, and 0.1% Tween 20 for 1 h at room temperature, probed first with specific antibodies and then with appropriate secondary antibodies. Bound antibodies were detected using the Novex ECL, HRP Chemiluminescent Substrate Reagent Kit (Life Technologies). Filters were autoradiographed, and images were acquired through the BioSpectrum Imaging System (Ultra-Violet Products Ltd., Cambridge, UK). The following antibodies were used: UCP2 (sc-6525), *β*-actin (sc-1615), HKII (ab-76959), MCT-4 (376140), GLUT1 (ab-209449), SOX2 (sc-16320), total OXPHOS (ab-110143), and *β*3-AR (h-160).

### 2.5. Metabolic Assays

The lactate extrusion assay was performed using the Lactate Colorimetric Assay Kit II, following the manufacturer's instructions (BioVision®).

The glucose uptake assay was performed using the Glucose Uptake Cell-Based Assay Kit (item n. 600470), following the manufacturer's instructions (Cayman Chemicals®).

ATP production assay was performed using the ATPlite Luminescence Assay System, following the manufacturer's instructions (PerkinElmer®).

All experiments were collected after 48 h, and the luminescence and fluorescence were measured with a Flex spectrophotometer.

### 2.6. ROS Cytofluorimetric Analysis

The cells were stained with 1 *μ*l of MitoSOX and were incubated for 15 minutes, then washed with PBS one time; the supernatant was aspirated; and the cells were incubated with Accutase. Finally, the detached cells were suspended in a final volume of 300 *μ*l and were analyzed using MACSQuant FACS (Miltenyi Biotec).

### 2.7. DilC(5) Membrane Potential

Membrane potential was performed using the MitoProbe™ DiIC1(5) Assay Kit for Flow Cytometry M34151, following the manufacturer's instructions (Thermo Fisher®).

### 2.8. Frap Assay

The ferric reducing antioxidant power (FRAP) assay, which is a measure of the antioxidant effects of nonenzymatic defences in plasma, was performed according to the method by Benzie and Strain [31].

### 2.9. Confocal Microscopy

After washing with PBS, the cells were fixed with a 3.7% formaldehyde solution in PBS for 20 min at 4°C. Then, after extensive washes in PBS, the cells were permeabilized with 0.1% Triton X-100 in PBS and then stained with a 50 *μ*g/ml fluorescent phalloidin conjugate solution in PBS, phalloidin-TRITC, for 1 h at room temperature with anti-*β*3-AR antibody, MitoProbe, and DAPI, then analyzed with the related laser. After several washes with PBS, the coverslips were mounted with glycerol plastine and then observed under a confocal fluorescence microscope (Leica).

### 2.10. Mitochondrial Isolation

To isolate the mitochondria from A375 stem cells and ESC, the Mitochondria Isolation Kit (Miltenyi Biotec®) was used. At the end of the kit procedure, after the centrifugation at 13,000xg for 2 minutes at 4°C, we aspirated the supernatant and resuspended the mitochondria pellet in lysis buffer for Western blot analysis and in PBS for FACS analysis.

### 2.11. MTT Assay

Viability of tumor cells, in all conditions, was evaluated using an MTT (3-[4,5-dimethylthiazol-2-yl]-2,5-diphenyltetrazolium bromide; thiazol blue) assay. It is a test of cell viability based on the reduction of the MTT dye by mitochondrial succinate dehydrogenase enzyme, which is active only in living cells. The enzyme cuts the tetrazolium ring of MTT (yellow substance) with the formation of a blue salt that is exocitated and precipitates.

The cells were maintained in MTT for 1 h, then the formation of needle-like crystals near the membrane was visible (if the cell is not vital, the process is reduced). Then, the cell membrane was lysed and the dye solubilized developing a color whose intensity was proportional to cell viability. The absorbance was evaluated at 570 nm using a spectrophotometer.

### 2.12. Bioinformatic Analysis

To assess the bioinformatic analysis, we chose four widely used methods: Hum-mPLoc 3.0, FUEL-mLoc, WegoLoc, and COMPARTMENTS. All the selected predictors are available as Web servers that are intended for eukaryotic proteins or are specific for human proteins. These predictors take advantage from the usage of the Gene Ontology (GO) terms, representing gene product properties. GO-based methods use the correlation between the annotations (usually functional annotations) for a protein and its subcellular location. Methods such as FUEL-mLoc, WegoLoc, and Hum-mPLoc 3.0 adopt different databases. WegoLoc and COMPARTMENTS extract the GO terms from the UniProtKB database. Hum-mPLoc 3.0 uses the SWISS-PROT portal, and FUEL-mLoc adopts two newly created compact databases, namely, ProSeq and ProSeq-GO, that allow consuming much less memory and make predictions faster.

By unifying these information with evidence on protein localization from curated knowledge, high-throughput experiments, text mining, and sequence-based prediction methods, these tools infer on the subcellular localization of our *β*3-AR protein.

### 2.13. Statistical Analysis

In vitro data are presented as means ± standarddeviation (SD) from at least three experiments. Results were normalized versus control expression levels.

Statistical analysis was performed using GraphPad Prism software (GraphPad, San Diego CA, USA) by one-way analysis variance (ANOVA), followed by a Bonferroni post hoc analysis.

## 3. Results and Discussion

**Figure 1:**
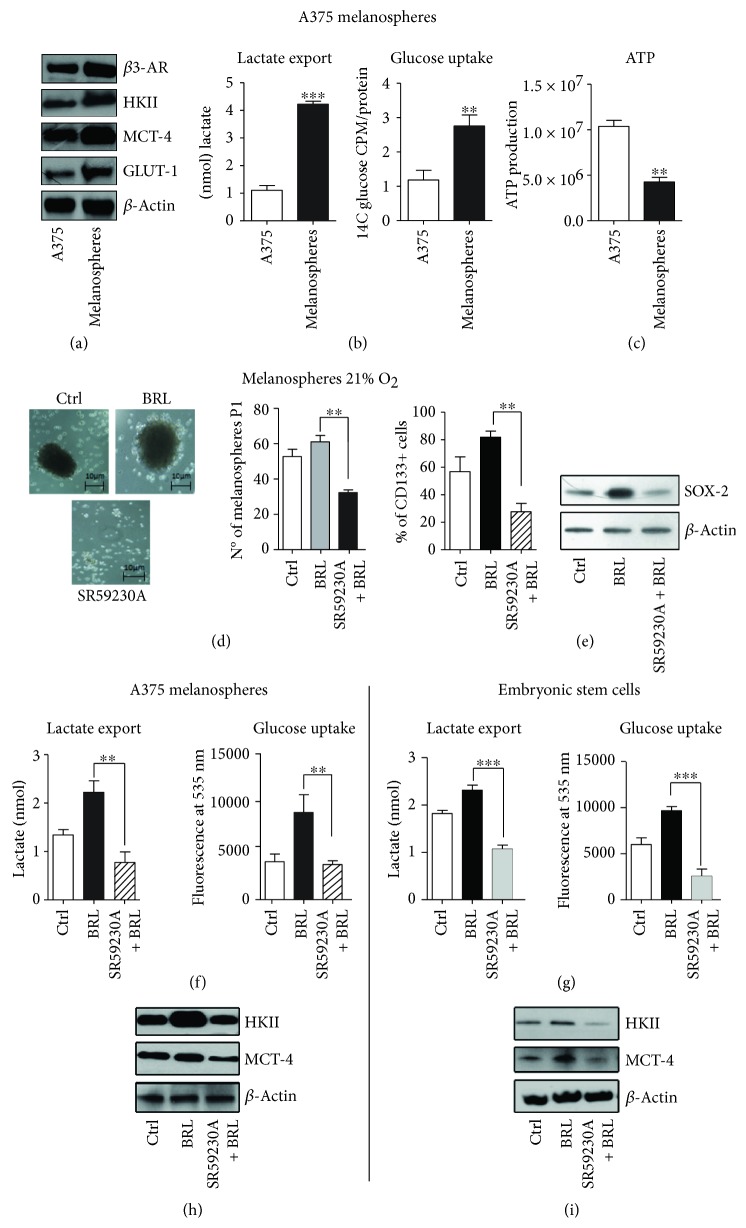
*β*3-AR affects the glycolytic metabolism of both A375 melanospheres and embryonic stem cell. WB analysis of *β*3-AR, hexokinase II (HKII), monocarboxylate transporter-4 (MCT-4), and GLUT-1 markers (a). Analysis of the lactate export and glucose uptake (b). ATP production assay (c). A375 melanospheres and mouse ES cells treated with BRL37344 (10 *μ*M) and BRL37344 (10 *μ*M) + SR59230A (10 *μ*M). The following experiments were performed: representative random figure of melanospheres in each condition and clonogenesis assay quantification (d); analysis of CD133^+^ cells by FACS and WB of stem cell marker SOX-2 (e); glucose uptake and lactate export in A375 melanospheres and ES cells (f, g); WB analysis of HKII and MCT-4 in A375 melanospheres (h) and ES cells (i). Data are representative of at least three experiments (mean and SD). ^∗∗^ *P* < 0.01 and ^∗∗∗^ *P* < 0.001.

Since embryos and cancer are enriched in nondifferentiated cells (embryonic and cancer stem cells), we investigated whether *β*3-AR affected CSC and ESC metabolism. We enriched the melanoma stem cell compartment by growing melanospheres *in vitro*. Interestingly, melanospheres revealed higher levels of *β*3-AR compared with A375 parental populations (Figure 1(a)). Usually, cells with an accelerated glycolysis rate display elevated glucose uptake, lactate overproduction, and overexpression of specific markers such as hexokinase 2 (HKII), monocarboxylate transporter-4 (MCT-4), and glucose transporter-1 (Glut-1) [32]. Here, we report that melanospheres expressed higher levels of HKII, MCT-4, and Glut-1 compared with the A375 parental population, thus demonstrating that A375 melanospheres rely on an accelerated glycolytic metabolism (Figure 1(a)). Elevated glycolytic metabolism in melanospheres was confirmed by a higher glucose uptake, lactate export, and decreased ATP synthesis, when compared with the A375 parental population (Figures 1(b) and 1(c)).

Selective *β*3-AR antagonism with SR59230A decreased the ability of A375 cells to form melanospheres under BRL37344 (selective *β*3-AR agonist) stimulations, as demonstrated by the reduction of P1 populations and reduction of stem cell markers CD133 and SOX2 (Figures 1(d) and 1(e)). To verify the metabolic effect of *β*3-AR stimulation, CSC and ESC were treated with BRL37344, inducing an accelerated aerobic glycolysis (Warburg effect), as confirmed by the increased expression of HKII and MCT-4, lactate export, and glucose uptake. These effects were dramatically reduced by SR59230A in both CSC and ESC (Figures 1(f)–1(i)). Supplementary Figure 1 shows that propranolol (unselective *β*1-/*β*2-AR antagonist) had no effect on the reduction of the Warburg metabolism in CSC. Thus, these results indicate that CSC and ESC share similar metabolic pathways (Warburg effect) and that this shift is mediated by *β*3-AR. The extrusion of lactate, promoted by *β*3-AR, enhanced the similarities between cancer and embryonic environment. During embryo development, the dramatic production and extracellular transport of lactate reduce the pH in the microenvironment, promoting the disaggregation of uterine tissues and facilitating the trophoblast invasion [11]. The lactate extrusion, together with the promotion of angiogenesis, cell migration, and metastatization, is probably also useful for the growth and infiltration of the tumor. Interestingly, lactate contributes significantly to the immune escape and further analogy between the tumor and the embryonic environment [33].

*β*3-AR's role has been well clarified in white and brown adipocytes. Selective, pharmacological activation of *β*3-AR has been shown to have strong effects on adipose tissue morphology and metabolism. The sympathetic nervous system, through *β*3-AR stimulations, is the main trigger of UCP1 induction and activation in brown fat mitochondria, leading to uncoupling of respiration and adaptive thermogenesis. UCP1, localized on the inner mitochondrial membrane, uncouples the activity of the respiratory chain from ATP synthesis, thereby releasing energy as heat [34]. Administration of CL-316,243, a potent and highly selective *β*3-AR agonist, leads to a marked increase in thermogenesis in brown adipose tissue (BAT) and an acute decrease in food consumption [35]. At the same time, the role of UCPs in cancer has been extensively studied, even though the effect of UCP2 on cellular energy balance in cancer cells remains unclear [23]. Current evidence demonstrates a link between UCP2 and the Warburg effect. Colon cancer cells stably overexpressing UCP2 produce progressively more lactate than do control-transfected cells, indicating higher rates of glycolysis [36]. Leukemia cells overexpressing UCP2 increase lactate production, decreasing the entry of pyruvate into the Krebs cycle, thereby inducing the Warburg effect [26, 27]. To assess a possible involvement of UCP2 in the *β*3-AR-induced Warburg effect, we used SR 59230A (specific *β*3-AR antagonist) and genipin (specific UCP2 inhibitor) under BRL37344 stimulations, in both CSC and ESC. UCP2 Western blotting expression analysis and lactate export assay at different concentrations of genipin were performed to evaluate which of them could have a similar effect to SR59230A treatment (Supplementary Figure 2).

**Figure 2:**
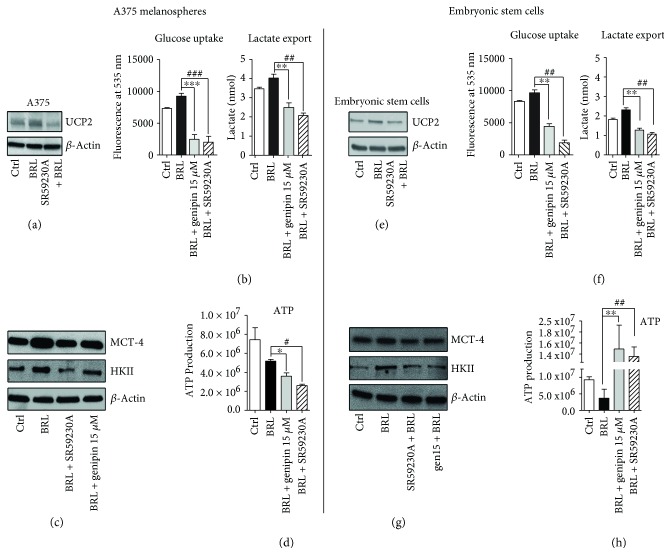
*β*3-AR/UCP2 axis blockade affects the glycolytic metabolism of both A375 and ES cells. Western blot analysis of UCP2 marker after treatment with BRL37344 (10 *μ*M) + genipin (15 *μ*M), BRL37344 (10 *μ*M), and BRL37344 (10 *μ*M) + SR59230A (10 *μ*M) (a, e). Glucose uptake and lactate export assays (b, f). WB analysis of HKII and MCT-4 markers (c, g). ATP production assay (d, h). Data are representative of at least three experiments (mean and SD). *P* values for SR59230A treatment ^∗^ *P* < 0.05, ^∗∗^ *P* < 0.01, and ^∗∗∗^ *P* < 0.001. *P* values for genipin treatment: ^#^ *P* < 0.05, ^##^ *P* < 0.01, and ^###^ *P* < 0.001.

Intriguingly, we demonstrated that SR59230A inhibited UCP2 expression in both CSC and ESC (Figures 2(a) and 2(e)). Performing functional metabolic assays, we observed that genipin, as well as SR59230A, decreased the lactate export and glucose consumption induced by BRL37344, both in CSC (Figure 2(b)) and in ESC (Figure 2(f)). Moreover, HKII and MCT-4 were impaired by genipin treatment (Figures 2(c) and 2(g)). The results obtained with genipin were comparable with those obtained with SR59230A, suggesting that CSC and mouse ESC share an accelerated *β*3-AR/UCP2-mediated glycolytic pathway.

Interestingly, the results regarding ATP production were not similar in ESC and CSC: the treatment with SR59230A and genipin induced a reduction in ATP synthesis only in A375 CSC but not in ESC, where ATP synthesis dramatically increased, indicating that only embryonic cells are able to shift to an aerobic metabolism (Figures 2(d) and 2(h)). These data confirm the hypothesis that *β*3-AR plays a crucial role in mitochondria UCP2 function and also highlight a different regulation of the *β*3-AR/UCP2 axis in CSC and ESC.

**Figure 3:**
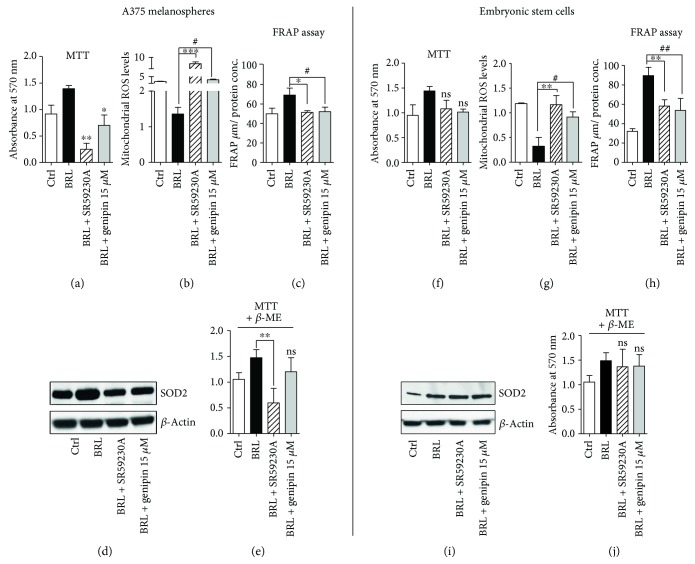
The antioxidant ability promotes survival in embryo with respect to tumor. The A375 melanospheres and ES cells were treated as in Figure 1. MTT survival experiment on each condition (a, f), mitochondrial mtROS measurement (b, g), FRAP assay (c, h), WB analysis of SOD-2 antioxidant marker (d, i), and MTT survival experiment on each condition in the presence of 100 *μ*M *β*-ME (e, j). Data are representative of at least three experiments (mean and SD). NS: not significant. *P* values for SR59230A treatment: ^∗^ *P* < 0.05, ^∗∗^ *P* < 0.01, and ^∗∗∗^ *P* < 0.001; *P* values for genipin treatment: ^#^ *P* < 0.05 and ^##^ *P* < 0.01.

The similarity in the impairment of glycolytic metabolism between the two cell lines and the difference in ATP production suggest a diverse regulation of cell survival pathways mediated by *β*3-AR. It has already been shown that SR59230A impairs cancer cell viability by inducing cytochrome C release [16], and this study shows that SR59230A and genipin dramatically reduced cell viability only in CSC (Figures 3(a) and 3(f)). Since UCP2 is implicated in mitochondrial ROS (mtROS) content modulation [37], we revealed that SR59230A and genipin increased mtROS content in both CSC (Figure 3(b)) and ESC (Figure 3(g)) treated with BRL37344, and the increase was markedly relevant in CSC (Figure 3(b)). Notably, the basal mtROS levels were definitely lower in ESC (Figure 3(g)) than in CSC (Figure 3(b)). This effect suggests a possible different antioxidant ability between the two cell lines. Results of this study indicate that the antioxidant ability induced by BRL37344 is definitely higher in ESC than in CSC, as confirmed by an increased FRAP functional assay in both cell lines (Figures 3(c) and 3(h)) [31]. In ESC, neither treatment with SR59230A nor treatment with genipin reduced SOD2 expression, if compared with CSC (Figures 3(d) and 3(i)). Therefore, after SR59230A and genipin treatment, ESC maintained a higher antioxidant ability compared to CSC, and this difference might explain the different effect on cell viability.

The addition of the ectopic antioxidant *β*-mercaptoethanol (*β*-ME) in the culture media recovered cancer cell death mediated by SR59230A and genipin in CSC (Figures 3(e) and 3(j)). These data demonstrate that the reduced viability of CSC after these treatments was due to their lower ability to counteract mtROS. In fact, the inhibition of the UCP2-mediated mtROS modulation ability in the CSC was critical for the survival of the A375 cell line. The antagonism of *β*3-AR dramatically increased A375 cell death compared to embryonic stem cells, demonstrating a strong selectivity for cancer cells. SR59230A, by inhibiting the *β*3-AR/UCP2 axis, strongly increases mtROS levels mainly in CSC, due to their inability to maintain antioxidant response compared to ESC. These results confirm data already present in literature, where *β*3-ARs and UCP2 are indicated to have a strong antioxidant role [37, 38]. The expression of *β*3-ARs and UCP2 in both cancer and embryonic stem cells is not surprising. Both types of cells need a strong ability to proliferate and strong protection from reactive oxygen species and present a highly *β*3-AR expression.

This result is in line with previous studies demonstrating a potent anticancer agent of the specific UCP2 inhibitor genipin [39]. Our data suggest that this selective ability to induce death only in CSC is due to a dramatic inability of tumor stem cells to maintain the same antioxidant response present in ESC. Literature reports that UCPs are directly activated by reactive oxygen species resulting in a negative feedback controlling both ROS production and their levels [37].

Recently, it has been shown that CSC have a markedly glycolytic profile with a decreased or dormant mitochondrial function, although the CSC phenotype can change between different cancer subtypes [32, 40]. Our results clearly suggest that the *β*3-AR/UCP2 axis promotes mitochondrial dormancy by inhibiting ATP production and mtROS content and leading both cell lines to increase aerobic glycolysis. Moreover, *β*3-AR antagonism promotes mitochondrial reactivation by inhibiting UCP2 activity and by increasing mtROS content.

There is accumulating evidence supporting a direct link between mitochondria, oxidative stress, and cell death. With the increasing number of newly discovered protein sequences in the postgenomic era, computational methods are required to address large-scale proteomics data and to establish new lines of experimental inquiry. Therefore, predicting subcellular location using computational tools has become a valuable approach for experimental validation.

At present, several public subcellular localization predictors are available. In our study, we chose four widely used methods: Hum-mPLoc 3.0, FUEL-mLoc, WegoLoc, and COMPARTMENTS. By unifying this information with evidence on protein localization from curated knowledge, high-throughput experiments, text mining, and sequence-based prediction methods, these tools infer on the subcellular localization of a protein.

In order to support our previous observation, we performed a bioinformatic analysis by using the aforementioned Web tools. The results shown in Supplementary Figure 3(a) represent the sum of ranked probability, estimated by each method. Surprisingly, all predictors we used designate the mitochondria as a potential candidate. As expected, the organelle does not appear as the best hit. This result is partly attributable to the different total number of proteins located in the different compartments; e.g., the plasma membrane contains more proteins than the mitochondria. A closer look at these results also shows that COMPARTMENTS assigns the best score to the mitochondria. This result is supported by the text mining *Z*-score computed by COMPARTMENTS, which designates the mitochondrion as the second best hit (Supplementary Figure 3(b)).

**Figure 4:**
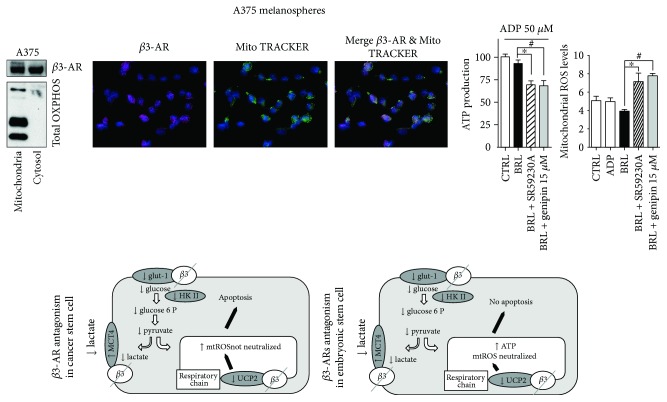
Functional *β*3-AR in mitochondria: a new receptor for an old compartment. WB analysis of *β*3-AR on mitochondria proteins (a). Confocal representative images of *β*3-ARs, MitoTracker, and merge of both markers (b). ATP production measured on isolated mitochondria as in Figure 1(d). Ectopic ADP was added to the reaction mix (c). mtROS measured as in Figure 3(a) (d). Summary scheme of the study (e). Data are representative of at least three experiments (mean and SD). *P* values for SR59230A treatment: ^∗^ *P* < 0.05; *P* values for genipin treatment: ^#^ *P* < 0.05.

Surprisingly, we found that *β*3-AR is expressed on mitochondria of both ESC (Supplementary Figure 3(c)) and CSC, as shown in the WB analysis and in the confocal images (Figures 4(a) and 4(b)). Moreover, through functional analysis, we demonstrated that SR59230A treatment is able to modify ATP production and mtROS levels in the isolated mitochondria (Figures 4(c) and 4(d)).

In summary, this study highlighted the parallelism between embryonic and cancer stem cells in the regulation of the *β*3-AR/UCP2-mediated Warburg metabolism and enhanced the different ability of the two cell lines to respond to SR59230A that induced cell death only in CSC by inhibiting antioxidant response (Figure 4(e)). An important result of this study is the presence of *β*3-AR in the mitochondria, suggesting a possible cooperation with *β*3-AR located in the plasmatic membrane. We suggest that *β*3-AR could work as sensor of redox state of cells directly influencing mitochondria bioenergetic functions. The presence of *β*3-AR in isolated mitochondria could be explained by its protective role against the induction of apoptosis, in both cancer and embryonic stem cells. Here, we clearly demonstrated a functional role of *β*3-AR blockade in isolated mitochondria by its ability to decrease ATP production and to increase mtROS levels.

Further experiments need to be performed to better clarify and elucidate the role of *β*3-AR in mitochondria, but the results of this study have shown a new and different possible function of this receptor in a new compartment.

Finally, we report that SR59230A is extremely selective in reducing the viability of CSC by blocking the Warburg metabolism and by inducing high mtROS levels. These results suggest a potential role of SR59230A as a strong and selective agent that could be used in cancer therapy.

## Data Availability

All data generated or analyzed during this study are included in this article (and its supplementary files). Requests for material should be made to the corresponding author.

## Supplementary Materials

Supplementary Materials Supplementary Figures: (1) metabolic assay of propranolol treatment; (2) metabolic assay with dose/response of genipin; (3) bioinformatic analysis of *β*3-AR presence prediction in different cell compartments.
