# Supplementary material for: β3-Adrenoreceptors Control Mitochondrial Dormancy in Melanoma and Embryonic Stem Cells
Source: Oxid Med Cell Longev. 2018 Nov 13;2018:6816508. doi: 10.1155/2018/6816508 (PMC6258109; doi:10.1155/2018/6816508)
Supplement: Supplementary Materials — Supplementary Figures: (1) metabolic assay of propranolol treatment; (2) metabolic assay with dose/response of genipin; (3) bioinformatic analysis of β3-AR presence prediction in different cell compartments. [file 6816508.f1.pdf]

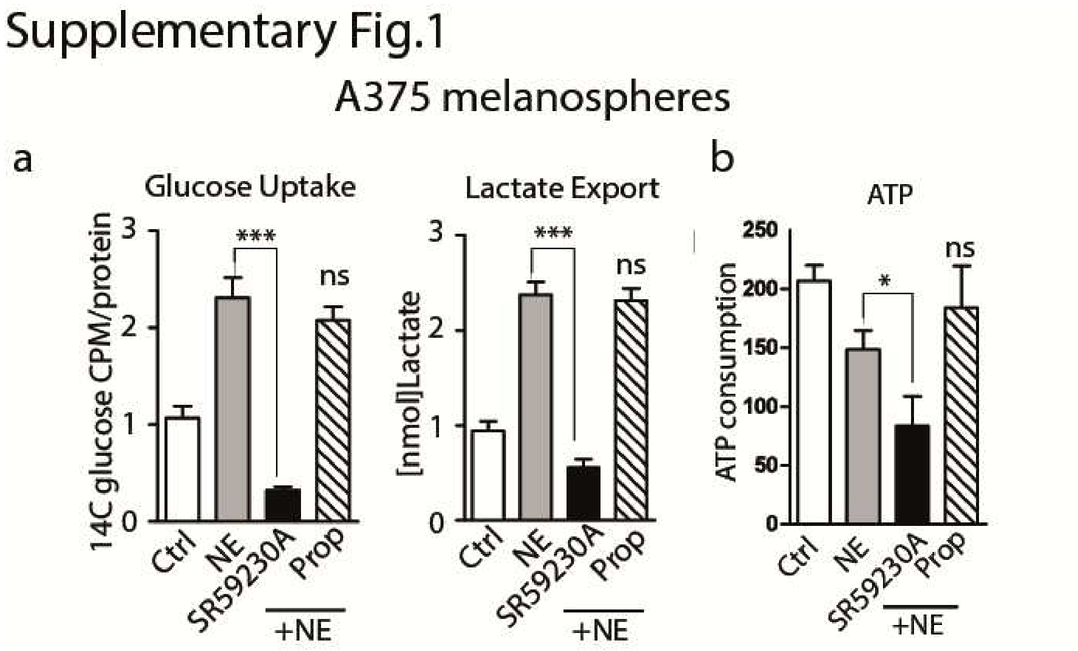

Supplementary Figure 1

Glucose uptake and Lactate export Assays (a) and ATP production Assay (b) in A375 Melanospheres treated with Norepinephrine (10µM), Norepinephrine (10µM) + Propranolol (10µM), Norepinephrine (10µM) + SR59230A (10µM). *P<0.05, ***P<0.001.

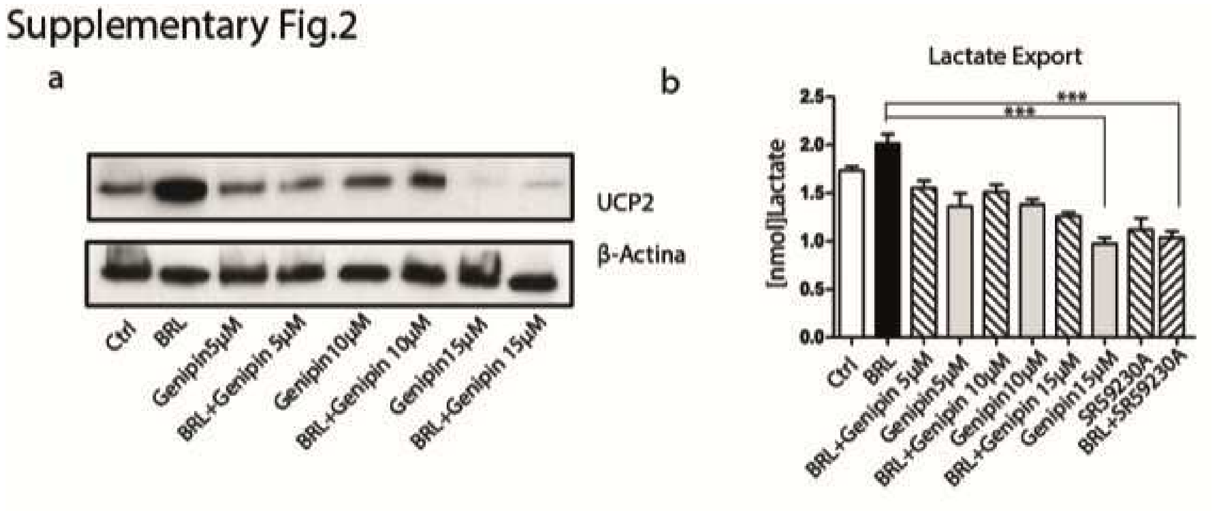

Supplementary Figure 2

WB analysis of UCP2 (a) and Lactate export Assay at different concentration of Genipin (b) in A375 Melanospheres treated with BRL37344 (10μM), BRL37344 (10μM) + different concentration of Genipin (5,10,15μM) and SR59230A at 10μM. ***P<0.001.

Supplementary Fig.3

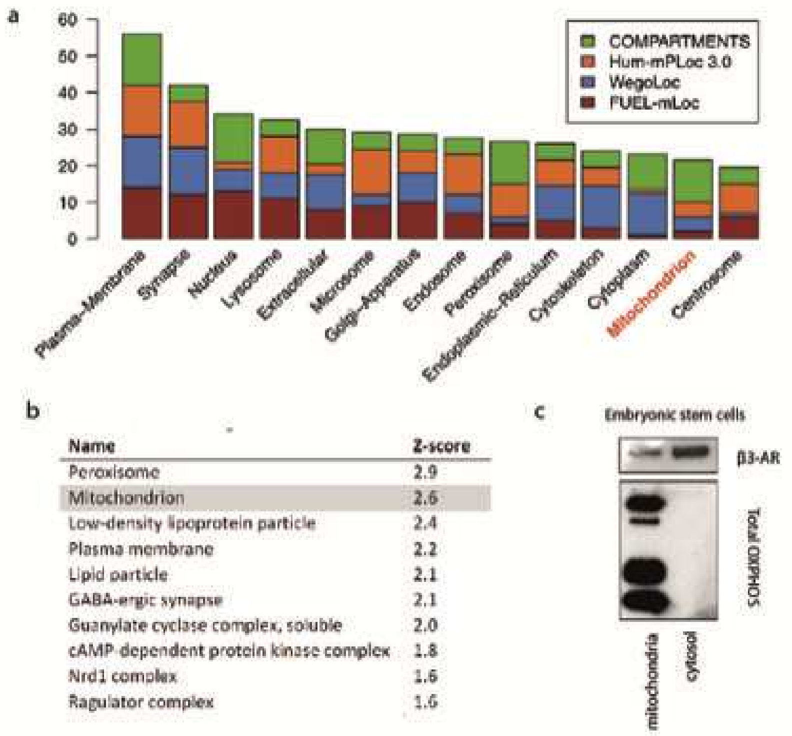

Supplementary Figure 3

Bioinformatic analysis of the β3-AR presence prediction in different cell compartments by database (a) and Z-score by Text-Mining (b). WB analysis of β3AR expèression in the mitochondria of ESC (c). Data represent at least three experiments (mean and SD).
